# Advanced gastroesophageal junction adenocarcinoma with skin involvement: a multidisciplinary perspective

**DOI:** 10.1093/omcr/omaf274

**Published:** 2026-02-18

**Authors:** Kholoud Alqasem, Sakhr Alshwayyat, Salsabeel Aljawabrah, Lara Alfrajat, Rand Al-atiyat, Raneem Hajjaj, Alaa BanyAmer, Mohammad Abu Shattal, Marwa Al-Shatti, Mohammed Al-mahdi Al-kurdi

**Affiliations:** Department of Internal Medicine, King Hussein Cancer Center, Amman, Jordan; Research Center, King Hussein Cancer Center, Amman, Jordan; Princess Basma Teaching Hospital, Irbid, Jordan; Applied Science Research Center, Applied Science Private University, Amman, Jordan; School of Medicine, The University of Jordan, Amman, Jordan; School of Medicine, The University of Jordan, Amman, Jordan; School of Medicine, The University of Jordan, Amman, Jordan; School of Medicine, The University of Jordan, Amman, Jordan; School of Medicine, The University of Jordan, Amman, Jordan; Department of Radiology, King Hussein Cancer Center, Amman, Jordan; Department of Pathology and Laboratory Medicine, King Hussein Cancer Center, Amman, Jordan; University of Aleppo, Faculty of medicine, Aleppo, Syrian Arab Republic

**Keywords:** Adenocarcinoma, Esophagogastric Junction, Palliative Care, Prognosis, Skin Metastasis

## Abstract

**Background:**

Skin metastasis secondary to visceral malignancy is a rare but poor prognostic occurrence. We report a rare case of gastroesophageal junction (GEJ) adenocarcinoma with skin metastases, highlighting the challenges in managing such cases.

**Case Presentation:**

A 63-year-old male diagnosed with stage IV Siewert 3 GEJ adenocarcinoma with initial extensive skin metastases. Palliative radiation and four cycles of first-line FOLFOX resulted in progression and new skin lesions. A second-line FOLFIRI and third-lines taxane regimens failed to yield clinical improvement and stop progression. His disease worsened with widespread metastases including omentum and peritoneum. He died 9 months after initial presentation due to intestinal perforation secondary to neutropenic fever.

**Conclusion:**

Skin metastases from GEJ adenocarcinoma are associated with aggressive disease chemotherapy resistance. A multidisciplinary approach, palliative care, and novel strategies are essential for managing such cases.

## Graphical Abstract

**Graphical Abstract:**
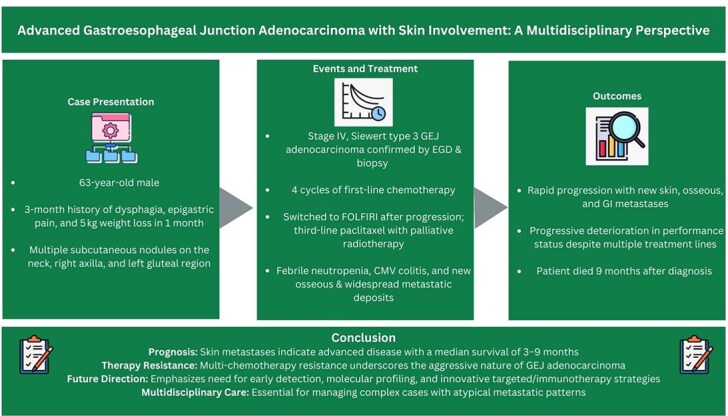

## Introduction

Visceral organs malignancies can have diverse cutaneous manifestations, ranging from non-malignant to malignant skin disorders. Malignant skin lesions affect 1-10% of cases, it can develop through several mechanisms, including hematogenous or lymphatic systemic spread, loco-regional spread, deposition of circulating tumor cells, or iatrogenic spread following procedures [1–4]. It carries a variable spectrum of presentations and usually indicates end-stage disease and poor prognosis [4, 5].

Cutaneous metastasis from gastric cancer is uncommon, occurring in only 0.8% to 1% of cases [3]. We report a case of 63-year-old male with advanced-stage GEJ adenocarcinoma and extensive subcutaneous metastases complicated by multi-chemotherapy resistance. The patient had a poor prognosis and eventually died nine months after the diagnosis.

## Case presentation

The patient is 63-year-old married male, smoker (60 pack-year) with a history of gastroesophageal reflux disease and vascular diseases needing multiple percutaneous interventions.

He presented on March 2023, with a three-month history of solid food dysphagia, unintentional 5-kg weight loss, epigastric pain and bloating, with noticed progression of symptoms over the last month. He complained of bilateral arm pain (predominantly on the right side), low back pain, and neck and axillary masses, which had become noticeable in the last few weeks. Upon physical examination, multiple skin lesions were noted on the lower neck, lateral aspect of the right axilla in the periscapular region and left gluteal region. These lesions were hard subcutaneous raised nodules with erythematous bases. The Eastern Cooperative Oncology Group (ECOG) performance status was 0. The family history was unremarkable for malignancy.

### Diagnosis of primary cancer

**Figure 1:**
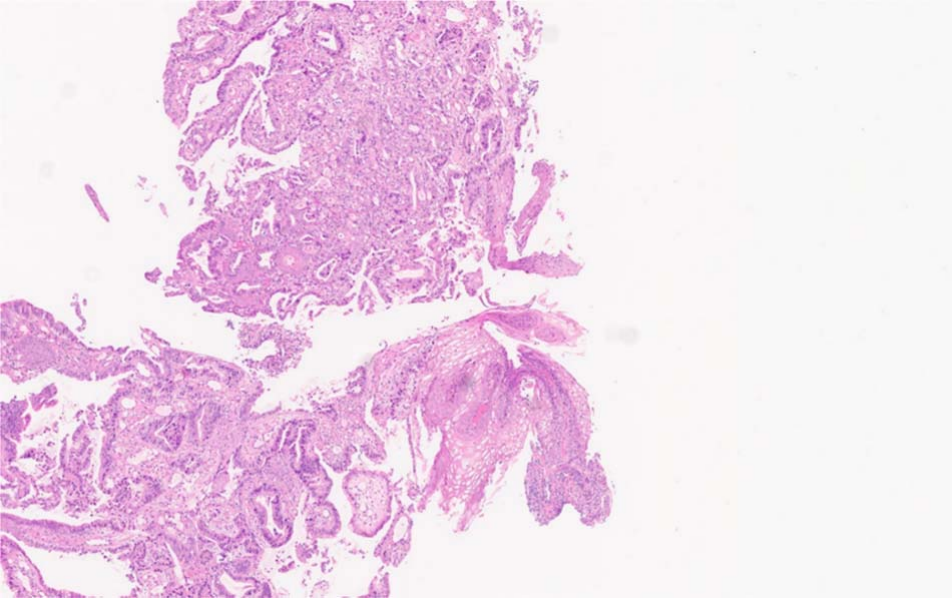
Gastroesophageal biopsy from the primary tumor stained with Hematoxylin and Eosin (H&E) showing moderately-differentiated adenocarcinoma.

Upper endoscopy revealed a tumor starting at 36 cm from the incisura with the GEJ identified at 41 cm. It involved the cardia and extended along the lesser curvature up to 46 cm. These findings were suggestive of Siewert 3 GEJ tumor. A biopsy showed *HER2* wild-type moderately differentiated adenocarcinoma (Figure 1).

### Staging workup

**Figure 2:**
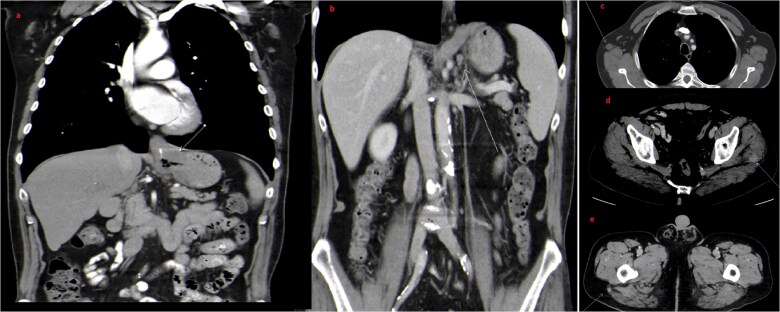
Coronal (a and b) and axial (c, d and e) images of initial staging contrast enhanced CT scan of the thorax, abdomen and pelvis. There is massive irregular GE junction thickening suggestive of a primary gastroesophageal tumor (arrow in a) as well as few regional lymph nodes (arrow in b). Also, There are few scattered intramuscular and subcutanous metastatic nodules (arrows in c, d and e).

**Figure 3:**
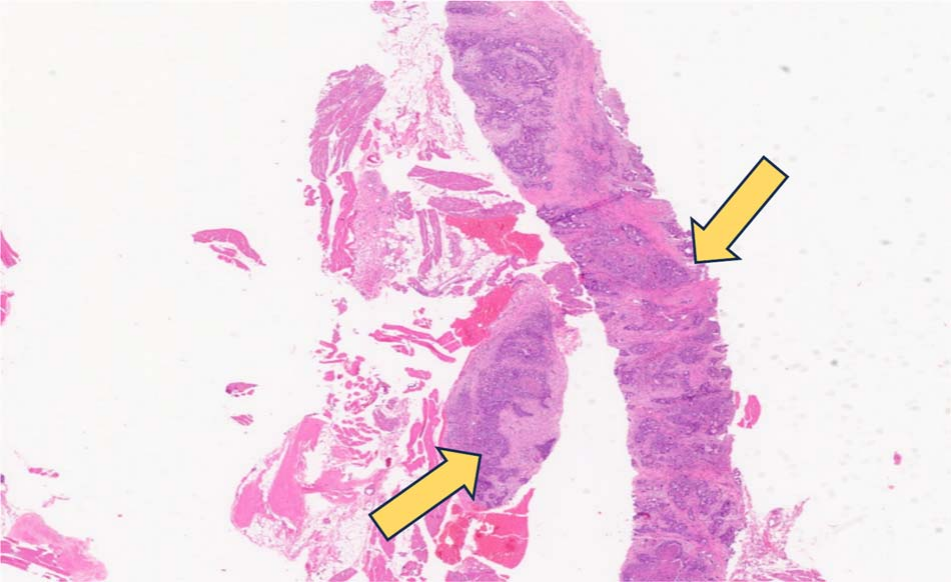
Gluteal skin biopsy (subcutaneous tissue) stained with Hematoxylin & Eosin (H&E) showing features of metastatic adenocarcinoma under low power magnification. The section shows dermis and subcutis infiltrated by atypical gland-forming epithelial structures with nuclear atypia and desmoplastic stromal reaction, consistent with metastatic adenocarcinoma (red arrow).

**Figure 4:**
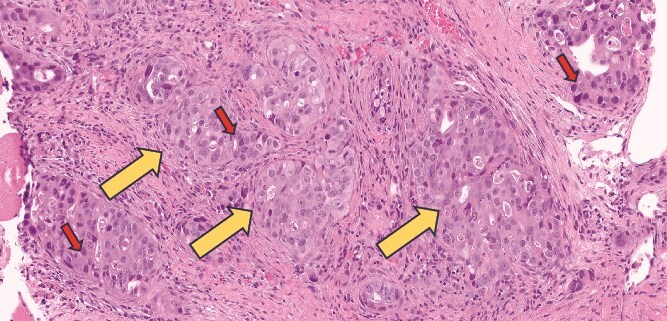
Gluteal skin biopsy (subcutaneous tissue) stained with Hematoxylin & Eosin (H&E) showing metastatic adenocarcinoma under high power magnification. (Red arrows) highlight the abnormal, irregularly shaped glands seen embedded in the subcutaneous tissue confirming the metastatic origin of the skin lesion. Nuclei appear hyperchromatic and crowded confirming nuclear atypia (red arrows).

A CT scan of the chest, abdomen, and pelvis, revealed circumferential wall thickening at the GEJ extending to the lesser curvature (Figure 2a, b, c, d and e). Small enhancing subcutaneous lesions were found in the right axilla and left gluteal subcutaneous/muscular tissues, raising suspicion of metastases. MRI of the head, chest, abdomen, and pelvis confirmed malignant gluteal nodules (largest measuring 1.3 × 1.9 cm). MRI has excluded the presence of any bone or brain metastatic lesions. Biopsy of the left gluteal mass confirmed metastatic adenocarcinoma (Figure 3 and 4). The final diagnosis was stage IV Siewert type 3 moderately differentiated GEJ adenocarcinoma with metastases to lymph nodes, skin, and subcutaneous/muscular tissues.

### Treatments and follow-up

The rarity and poor prognosis of the condition were discussed with the patient, who actively participated in decision-making throughout the disease course.

Despite initial improvement, disease progression was observed after 4 cycles of FOLFOX (oxaliplatin, 5-flurouracil, leucovorin), manifested with new skin metastases and worsening shoulder pain. A single axillary lesion showed a partial response. Given progression, chemotherapy was switched to FOLFIRI (irinotecan, leucovorin, and 5-fluorouracil).

**Figure 5:**
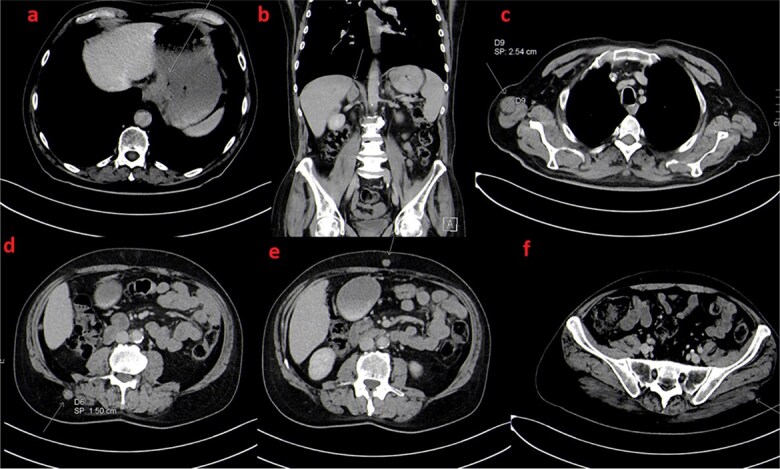
Axial (a, c, d, e and f) and Coronal (b) images from follow up CT scan after 5 months again demonstrate GE junction primary tumor (a). Also, there is progression in the size of many preexisting muscular and subcutaneous deposits (c) in addition to development of new lesions (d, e and f) as well as adrenal metastasis (b).

After one cycle, new osseous metastases in the right humerus and soft tissue deposits were identified. Palliative radiotherapy was administered, but pelvic MRI revealed additional gluteal and femoral metastases. Following 4 cycles of FOLFIRI, subcutaneous and intramuscular metastases increased (Figure 5). A third-line chemotherapy (paclitaxel) and palliative radiotherapy to painful lesions were initiated. The patient developed febrile neutropenia and cytomegalovirus colitis after the third paclitaxel dose. Imaging confirmed new gastrointestinal and thoracic metastases. Despite interventions, pain worsened due to further bony metastases. CT imaging demonstrated further progression of subcutaneous and soft tissue metastatic deposits in the neck, thorax, abdomen, and pelvic regions. The patient’s performance status continued to deteriorate, and palliative radiotherapy was administered to the left neck and left arm.

### The outcome

**Figure 6:**
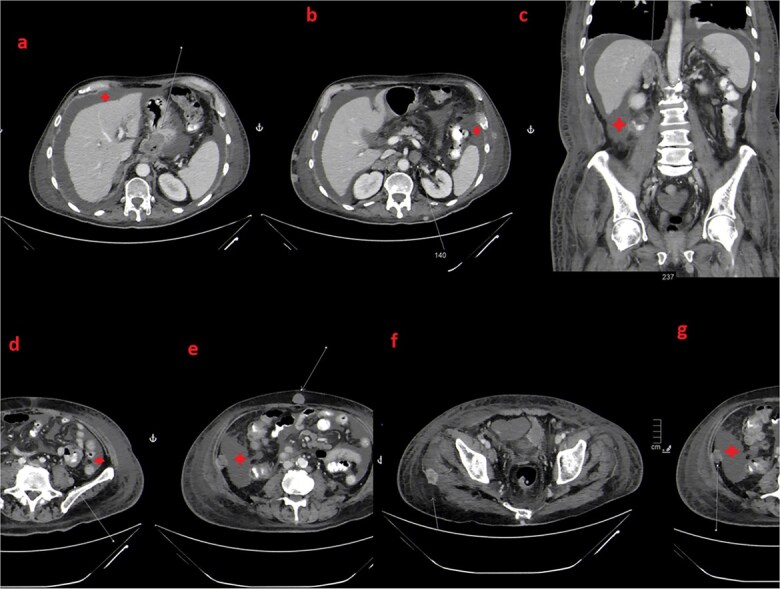
Axial (a, b, d, e, f and g) and Coronal (b) images from follow up CT scan after 4 months show progression of the GE junction primary tumor (a), right adrenal metastasis (c) and many preexisting subcutaneous and muscular deposits (e). Also, there is the development of new ascites (star), peritoneal deposits (g) and other muscular and subcutaneous lesions (d and f).

The patient presented to the emergency room with sudden lower abdominal pain and unstable vital signs consistent with shock. CT imaging revealed bowel perforation with pneumoperitoneum and pelvic ascites. There was generalized mucosal thickening of the jejunal walls, along with progression of the GEJ tumor and increased metastatic peritoneal, omental, and intramuscular nodules (Figure 6). The patient was admitted to the ICU with septic shock. The prognosis was extremely poor, with signs of metabolic acidosis, high anion gap, and acute liver injury. Surgical intervention was deemed unfeasible, and palliative care was initiated. The patient died from the disease on December, 2023.

## Discussion

Gastroesophageal cancer skin metastasis accounts for less than 1% of cases [3]. We reported a patient with stage IV GEJ adenocarcinoma presented with extensive cutaneous metastases. Highlighting the importance of considering skin lesions as a potential initial manifestation of internal malignancies. Despite multiple lines of chemotherapy, our patient demonstrated treatment resistance and eventually died nine months after diagnosis.

We conducted a comprehensive review aimed to reveal the clinical presentation, treatment outcomes and resistance profile of skin metastases in gastroesophageal cancer to underscore the atypical nature, the predominance of poor prognostic factors and drug resistance among the patients (Supplementary Table 1).

Molecular profiling reveals mechanisms of chemotherapy resistance, including dysregulated long non-coding RNAs, overexpression of the EGFR pathway (e.g., PI3K/Akt/mTOR), epithelial-mesenchymal transition, enhanced DNA repair capabilities (e.g., ERCC1 expression), stromal invasion, altered architecture, molecular heterogeneity between the primary tumor and skin metastases, and the adaptive cellular functioning and microenvironment which eventually lead to the resistance [6–9]. Unfortunately, molecular testing for genes other than HER2 was not considered due to the limited resources and accelerated deterioration of the case which hindered the understating of disease nature and the possibility of targeted therapies in our patient.

Immunotherapy resistance mechanisms include genetic mutations affecting antigen presentation, signaling pathway abnormalities (e.g., IFN-γ, Wnt/β-catenin), and an immunosuppressive tumor microenvironment rich in regulatory T cells and myeloid-derived suppressor cells [10]. While these agents hold promise, their benefits in patients with widespread skin metastases, as in our case, remain uncertain.

Cutaneous metastases in visceral organ malignancies, particularly those with gastrointestinal origin, remains a challenging scenario given the lack of global guidelines, limited reported cases and failure of disease control after the systemic treatments. However, in cases of limited skin involvement, local treatment could demonstrate feasibility with relatively better outcomes. Surgical excision that follows the melanoma surgery protocol with 1 cm safety margin was found to be a successful approach in selected patients [11]. Other local interventions were recommended in palliative settings and patients not fit for surgery. Interventions like radiotherapy, laser ablation, cryotherapy and electrochemotherapy are continuously developed to treat those patients [12]. In our case, the patient was managed with systemic therapy due to the deteriorated performance status. However, the decision of the best treatment modality should be adjusted to the burden of disease, performance status and the benefit vs. risk balance in such patients.

While our case lacking proper comprehensive molecular and pathological testing due to the limited resources and retrospective nature of the study, next generation sequencing could provide a better understanding for disease biology while providing a precision approach for therapeutic decision [13, 14]. Genetic alterations like *BRAF* mutation, microsatellite instability (MSI) and PD-1 expression have been integrated in the NCCN management guidelines for advanced gastric cancer [15]. Those alterations were found to guide the biological processes of atypical aggressive presentations -such as skin metastasis- although representing an area for targeted therapy with superior survival benefits, e.g PD-1 and MSI-high gastric cancer were found to be a therapeutic biomarker for immunotherapy regimen, while *BRAF* was identified as a promising targetable mutation that influences the prognosis positively [16]. Expanding genetic testing for such cases should be recommended given the complex disease course and devastating outcomes.

## Conclusion

Skin metastases from internal malignancies indicate an advanced stage with poor prognosis. They are associated with treatment resistance and limited therapeutic options. This case underscores the necessity for early identification and the multidisciplinary approach to optimize patient outcomes and quality of life. Continued research in molecular profiling, targeted therapies, and immunotherapy, are crucial to improve survival.

## Supplementary Material

Skin_metastasis_Suplemmentary_Table_omaf274

## Data Availability

No datasets were generated or analysed during the current study.
