# Supplementary material for: Advanced gastroesophageal junction adenocarcinoma with skin involvement: a multidisciplinary perspective
Source: Oxf Med Case Reports. 2026 Feb 18;2026(2):omaf274. doi: 10.1093/omcr/omaf274 (PMC12916007; doi:10.1093/omcr/omaf274)
Supplement: Skin_metastasis_Suplemmentary_Table_omaf274 [file skin_metastasis_suplemmentary_table_omaf274.docx]

| **Authors (Year)** | **Study Type** | **Study Title** | **Patient Demographics** | **Histology** | **Clinical Presentation** | **Treatment** | **Outcome** | **Conclusion** |
| --- | --- | --- | --- | --- | --- | --- | --- | --- |
| **Chemotherapy, Surgery & Supportive Care** | | | | | | | | |
| Yao et al. (2024)(1) |  | Cutaneous metastasis from gastric cancer: Manifestation, diagnosis, treatment and prognosis | 72 patients; Mean age 60; predominantly male | 87.5% poorly differentiated; 66.1% signet ring cell carcinoma | Nodular lesions (most common), erysipelas-like, and ulcerative lesions; thoracoabdominal wall (56.9%) and head/neck (54.2%) most affected sites; 64.7% had extracutaneous metastases | 65.6% chemotherapy; 18.8% surgery post-gastrectomy | Median survival: 6 months overall; 48 months with surgery (P = 0.001) | Skin metastases often indicate systemic disease; surgical intervention significantly improves survival in selected cases |
| Cesaretti et al. (2014)(2) | Case Report | Cutaneous Metastasis From Primary Gastric Cancer | 60-year-old man | Poorly differentiated diffuse gastric adenocarcinoma | Erythematous lesion on chest and abdomen | Surgery (relaparotomy and excision) | Disease-free at 40 months follow-up | Early surgical intervention can manage cutaneous metastasis effectively |
| Cokgezer et al. (2020)(3) | Case Report | Cutaneous Metastasis of Signet Cell Gastric Carcinoma | 75-year-old female | Signet-ring cell gastric adenocarcinoma | Asymptomatic scar-like lesion on epigastric area | Chemotherapy | Progression to additional metastases | Signet-ring cell carcinoma can present as skin lesions and requires aggressive management |
| Park et al. (2011)(4) | Case Report | Metastatic Gastric Adenocarcinoma Presenting as a Solitary Plaque on the Palm | 80-year-old male | Moderately differentiated gastric adenocarcinoma | Erythematous plaque on the palm | Chemotherapy | Death after one month post-diagnosis | Rare presentation on the palm indicates advanced disease |
| Früh et al. (2005)(5) | Case Report | Resection of Skin Metastases from Gastric Carcinoma with Long-Term Follow-Up | 60-year-old male | Adenocarcinoma of the cardia (Stage II) | Six isolated skin metastases on head and thigh | Surgical resection | Remission 7.5 years post-diagnosis | Exceptional long-term survival post-surgical intervention for isolated skin metastases |
| Garcia et al. (2024)(6) | Case Report | A Rare Case of Cutaneous Gastric Adenocarcinoma With Signet Ring Cell Features | 48-year-old male | Poorly differentiated adenocarcinoma with signet ring cell features | Expanding rash on chest, neck, and face | Chemotherapy | Treatment ongoing at time of report | Early recognition of cutaneous signs can lead to timely treatment |
| Koo et al. (2007)(7) | Case Report | Cutaneous metastasis resembling acute dermatitis in patient with advanced gastric cancer | 60-year-old female | Advanced gastric cancer with signet-ring cells | Erythematous papules, weeping crusts, and scales on cheeks, forehead, scalp, and nasolabial folds; initially misdiagnosed as contact dermatitis | Chemotherapy (capecitabine and cisplatin) | Complete clinical remission | Cutaneous metastasis may mimic dermatitis; biopsy and immunohistochemistry are crucial for accurate diagnosis. Cisplatin-based chemotherapy showed effective and sustained response in treating cutaneous lesions |
| Souza et al. (2020)(8) | Case Report | Signet ring cells in carcinomatous lymphangitis due to gastric adenocarcinoma | 72-year-old female | Poorly differentiated adenocarcinoma with signet ring cells | Lymphangitis and skin infiltration on lower limbs and abdomen | Palliative chemotherapy | Progression to respiratory failure and death | Rapid progression despite treatment, highlighting aggressive nature of disease |
| Frey et al. (2009)(9) | Case Report | Cutaneous metastases as the first clinical sign of metastatic gastric carcinoma | 54-year-old male | Gastric tubular adenocarcinoma | Multiple pea-sized scalp nodules | Polychemotherapy | Primary tumor regressed, scalp nodules disappeared | Unusual case of scalp metastasis from gastric cancer responding well to chemotherapy |
| Koyama et al. (2019)(10) | Case Report | Late Cutaneous Metastasis Originating from Gastric Cancer with Synchronous Metastasis | 89-year-old male | Gastric cancer (T3N0M1, pStage IV) | Small reddish nodules in the right axilla | En-bloc resection; No additional treatment | Alive without recurrence | Cutaneous metastases require meticulous follow-up for early detection |
| Demircioğlu et al. (2021)(11) | Case Report | Livedoid cutaneous metastasis of signet-ring cell gastric carcinoma | 53-year-old female | Signet-ring cell gastric carcinoma | Erythematous, reticular, livedoid patches on abdomen and thighs | Chemotherapy, local radiotherapy | Died in December 2018 | High index of suspicion needed for diagnosing cutaneous metastases |
| De Giorgi et al. (2019)(12) | Case Report | Appearance of cutaneous melanoma and subcutaneous metastases in metastatic gastric carcinoma undergoing chemotherapy | 61-year-old male | Gastric adenocarcinoma with melanoma | Multiple cutaneous metastases resembling sebaceous cysts | Systemic chemotherapy | Died from progressive disease | Immunotherapy and close dermatological follow-up recommended |
| Yasuda et al. (2020)(13) | Case Report | Recurrence of Cutaneous and Lymph Node Metastases 12 Years after Radical Total Gastrectomy for Stage IIA Gastric Cancer | 62-year-old male | Poorly differentiated adenocarcinoma | Jugular swelling, cutaneous and lymph node metastases | Chemoradiotherapy | Died 8 months post-diagnosis | Late recurrences are rare and require long-term vigilance |
| Chen et al. (2021)(14) | Case Report | Extensive cutaneous metastasis of recurrent gastric cancer | 69-year-old male | Recurrent gastric cancer | Widespread cutaneous metastases | Hospice care | Died 1 month later | Highlighting the severity and poor prognosis of extensive cutaneous metastases |
| Kurmus et al. (2024)(15) | Case Report | Cutaneous Metastases of Signet-Ring Cell Gastric Carcinoma | 70-year-old male | Signet-ring cell gastric carcinoma | Indurated plaques and erysipelas-like erythema | Chemotherapy | Poor prognosis due to aggressive nature | Importance of early detection and multidisciplinary approach |
| Yao et al. (2023)(16) | Case Report | A case of delayed cutaneous metastases from signet-ring cell mixed-type gastric cancer | 61-year-old male | Signet-ring cell mixed-type gastric cancer | Skin nodules on head, groin, thigh | Chemotherapy | Living but with ongoing treatment | Significance of multidisciplinary approach for skin lesions in cancer patients |
| Tian et al. (2023)(17) | Case Report | Inflammatory cutaneous metastases originating from gastric cancer | 65-year-old male | Poorly differentiated adenocarcinoma | Skin redness, swelling, nodules | Hospice care | Died 7 days after diagnosis of cutaneous metastasis | Cutaneous metastasis indicates poor prognosis and rapid progression |
| Makino et al. (1993)(18) | Case Report | A case of complete remission of metastatic skin carcinoma (erythema type) from advanced gastric cancer by CDDP administration | 75-year-old female | Moderately differentiated tubular adenocarcinoma (tub2), Borrmann type 3 | Erythematous skin metastases on chest and neck progressing to nodular type | Cisplatin (CDDP) 25 mg intermittently; prior regimens with 5-FU, MMC, and epirubicin ineffective | Complete response; lesions disappeared; CEA normalized; patient remained well for 3.5 years | Cisplatin (CDDP) can induce complete remission in cutaneous metastases resistant to other regimens; careful monitoring needed due to potential toxicity (thrombocytopenia, liver dysfunction) |
| Tajima et al. (1994)(19) | Case Report | A case of cutaneous and brain metastasis of gastric carcinoma, treated effectively by chemotherapy with CDDP, MMC, etoposide and 5'-DFUR | 67-year-old female | Poorly differentiated adenocarcinoma | Multiple cutaneous nodules on trunk and brain metastases (headache, CT-confirmed lesions) | Chemotherapy with CDDP, MMC, etoposide, and 5'-DFUR | Disappearance of skin and brain lesions after 3 cycles; patient died of lung metastasis 24 weeks later | Multidrug chemotherapy including CDDP and 5'-DFUR can induce significant short-term remission of cutaneous and CNS metastases from gastric cancer |
| Matsuoka et al. (2013)(20) | Case Report | A case of delayed subcutaneous metastases of gastric carcinoma effectively treated with S-1 plus cisplatin chemotherapy | 45-year-old female | Poorly differentiated adenocarcinoma; signet-ring cell carcinoma in metastatic sites | Delayed subcutaneous metastases (head and back) and ovarian tumor 11 years after surgery | S-1 + cisplatin (8 cycles); surgical resection of ovarian tumor | Cutaneous metastases disappeared; ovarian tumor progressed but was resected | S-1 + cisplatin therapy can effectively treat delayed subcutaneous metastases in gastric cancer; long-term surveillance is crucial due to potential for very late recurrence |
| Mezawa et al. (2003)(21) | Case Report | A case of gastric carcinoma with multiple skin, bone, and bilateral ovary metastasis; effective treatment by chemotherapy | 40-year-old female | - | Multiple metastases: skin, bone, and bilateral ovaries; initial symptom: general malaise | Weekly 5-FU and cisplatin; later CPT-11 alone after one cycle of CPT-11 + cisplatin | Survived 38 months after diagnosis | Weekly low-dose 5-FU/CDDP and CPT-11 can be effective and preserve quality of life in widespread metastatic gastric cancer, including cutaneous metastases |
| Morita et al. (2017)(22) | Case Report | A Case of Long-Term Survival of Cutaneous Metastasis from Primary Gastric Cancer | 65-year-old female | Well-differentiated adenocarcinoma (tub1), pStage IIA | Umbilical cutaneous metastasis and peritoneal dissemination 5 years after distal gastrectomy | S-1 + cisplatin (7 courses), then paclitaxel (33 courses) | Complete response; alive >3 years post-recurrence | Long-term survival is possible in cutaneous metastasis from gastric cancer; sequential chemotherapy with S-1, cisplatin, and paclitaxel can lead to durable complete remission |
| Matsuoka et al. (2021(23) | Case Report | A long-term survival case with recurrent esophageal adenosquamous carcinoma | 46-year-old male | Adenosquamous carcinoma of the esophagogastric junction; poorly differentiated adenocarcinoma with squamous component | One month post-esophagectomy: cutaneous metastasis (chest wall), adrenal metastasis, and bone metastasis (C2 vertebra); initial presentation: dysphagia, weight loss | Neoadjuvant FP (5-FU + cisplatin); surgery; recurrence treated with 27 cycles of weekly docetaxel and radiotherapy (36 Gy/16 Fr) to bone | Complete remission of cutaneous, adrenal, and bone metastases; disease-free for >8 years | Aggressive multimodal therapy (chemotherapy, surgery, radiotherapy) can achieve durable complete remission even in recurrent metastatic adenosquamous carcinoma. Immune response (CD8⁺ TILs, PD-L1 TPS >50%) may have contributed to long-term survival |
| **Immunotherapy** | | | | | | | | |
| Menghani et al. (2020)(24) | Case Report | Gastric Cardia Adenocarcinoma with Metastasis to the Scalp: A Case Report | 69-year-old male | Moderately differentiated gastric cardia adenocarcinoma (Siewert type III, cT3N0Mx) | Occipital scalp lesion (friable plaque with yellow scale and crusts); metastatic via dermal biopsy with lymphatic invasion; widespread disease on PET/CT (liver, bone, scalp) | Initial chemotherapy refused; after 20 months, started on pembrolizumab based on PD-L1 positivity (CPS = 1) | Died a few weeks after starting pembrolizumab due to extensive metastatic burden | Scalp metastases may indicate disseminated gastric cancer. Immunohistochemical profiling (e.g., PD-L1) is essential for guiding therapy, especially immunotherapy. Delayed standard care may limit therapeutic success despite targetable markers |
| Sai et al. (2021)(25) | Case Report | Advanced Gastric Cancer with Recurrence of Skin Metastasis 9 Years After Surgery | 70-year-old male | Adenocarcinoma (pT4a, pN3, M0, Stage IIIC) | Subcutaneous swelling in inguinal-scrotal region; later rectal stenosis due to peritoneal spread | Nab-paclitaxel + ramucirumab → nivolumab | Disease progression requiring colostomy | Late recurrence as skin metastasis is possible; sequential systemic therapies used, including immunotherapy |
| Hao et al. (2024)(26) | Case Report | Advanced Gastric Cancer with Multiple Skin Metastases Relieved by Immunotherapy | 61-year-old female | Poorly differentiated adenocarcinoma (Lauren diffuse type) | Cauliflower-like nodules on back, neck, chest, abdomen; erythematous patches | 10 cycles of immunotherapy (Tislelizumab 200mg) + chemo (albumin-bound paclitaxel 100mg) | Partial remission; significant skin improvement | Rare cutaneous metastases confirmed by IHC and genetic testing; immunotherapy (anti-PD-1) showed significant clinical benefit in metastatic gastric cancer with skin lesions |
| Zhang et al. (2022)(27) | Case Report | Cutaneous Metastasis from Esophageal Squamous Cell Carcinoma: A case report | 82-year-old male | Poorly differentiated squamous cell carcinoma | Abdominal wall: hard, rough, dark skin 13 months post-diagnosis | 4 cycles chemotherapy, then apatinib + docetaxel ×2, followed by nivolumab ×2 cycles | Died of multiple organ failure | Cutaneous metastasis is a rare and late manifestation of esophageal SCC; typically associated with poor prognosis and limited treatment efficacy |
| Ryu et al. (2021)(28) | Case Report | Scalp Metastasis of Advanced Gastric Cancer | 60-year-old male | Metastatic gastric adenocarcinoma | 1 cm fixed, skin-colored scalp nodule (2-year duration); prior Hx of stage IIB gastric cancer, gastrectomy 3 yrs earlier | Excision & biopsy of scalp nodule → metastatic adenocarcinoma confirmed; staging laparotomy revealed peritoneal, pancreatic, vascular, and liver metastases. Treated with chemotherapy: leucovorin, 5-FU, oxaliplatin, paclitaxel, ramucirumab | Patient died one year later following progression | Cutaneous metastasis can occur years after gastrectomy; scalp metastases, though rare, should raise suspicion for systemic recurrence in patients with prior gastric cancer history |
| **Targeted Therapy** | | | | | | | | |
| Han et al. (2021)(29) | Case Report | Case Report: Herceptin as a Potentially Valuable Adjuvant Therapy for a Patient with HER2+ Advanced Esophageal Squamous Cell Carcinoma | 61-year-old male | HER2-positive squamous cell carcinoma of the esophagus | Progressive dysphagia, diagnosed with non-resectable cervical and thoracic esophageal lesions; later, lung, skin, and brain metastases | Chemoradiotherapy followed by HER2-targeted therapy (trastuzumab + albumin-bound paclitaxel + cisplatin) | Partial remission with PFS ~6 months, OS ~19 months | HER2-targeted therapy with trastuzumab may be beneficial in HER2-positive esophageal squamous cell carcinoma, even with advanced or metastatic disease |
| Katayama et al. (2017)(30) | Case Report | Gastric Cancer Diagnosed with Metastasis of the Navel (Sister Mary Joseph's Nodule) – Case report | Female, 60s | Gastric adenocarcinoma | Pain and induration at the umbilicus; Sister Mary Joseph’s nodule; later chest skin metastasis | Total gastrectomy, umbilical mass excision, sigmoidectomy (due to peritoneal dissemination); chest skin tumor enucleation; 47 courses of capecitabine, cisplatin, and trastuzumab (HER2-targeted therapy) | No recurrence or metastasis 5 years post-op | Aggressive surgical intervention and prolonged systemic HER2-targeted chemotherapy can achieve long-term survival even in stage IV gastric cancer with skin metastases |

**Table 1 Supplementary:** Overview of Case Reports on Cutaneous Metastases from Gastric Cancer

**References**

1. Yao GL, Tao YJ, Fan YG. Cutaneous metastasis from gastric cancer: Manifestation, diagnosis, treatment and prognosis. European Journal of Surgical Oncology. 2024 Feb;50(2):107939.

2. Cesaretti M, Malerba M, Basso V, Boccardo C, Santoni R, Weiss A, et al. Cutaneous Metastasis From Primary Gastric Cancer: A Case Report and Review of the Literature Practice Points CUTIS Do Not Copy. 2014.

3. Cokgezer S, Samanci N, Bektas M, Kepil N, Demirelli F. Cutaneous metastasis of signet cell gastric carcinoma. Indian J Dermatol. 2020 Mar 1;65(2):148–50.

4. Park MK, Son IP, Park KY, Seo SJ. Metastatic gastric adenocarcinoma presenting as a solitary plaque on the palm. Ann Dermatol. 2011;23(SUPPL. 2).

5. Früh M, Ruhstaller T, Neuweiler J, Cerny T. Resection of skin metastases from gastric carcinoma with long-term follow-up: An unusual clinical presentation. Onkologie. 2005;28(1).

6. Garcia A, Maqsudlu A, Dhawa I, Chaaya A. A Rare Case of Cutaneous Gastric Adenocarcinoma With Signet Ring Cell Features. ACG Case Rep J. 2024 Mar;11(3):e01299.

7. Koo DH, Chang HM, Jung JY, Song JH, Lee JL, Ryu MH, et al. Cutaneous metastasis resembling acute dermatitis in patient with advanced gastric cancer. Clin Exp Dermatol. 2007 May;32(3):284–6.

8. Souza B da S, Bonamigo RR, Viapiana GL, Cartell A. Signet ring cells in carcinomatous lymphangitis due to gastric adenocarcinoma. An Bras Dermatol. 2020 Jul 1;95(4):490–2.

9. Frey L, Vetter-Kauczok C, Gesierich A, Bröcker EB, Ugurel S. Hautmetastasen als Erstes Klinisches Symptom Eines Metastasierenden Magenkarzinoms. JDDG - Journal of the German Society of Dermatology. 2009;7(10):893–5.

10. Koyama R, Maeda Y, Minagawa N, Shinohara T, Hamada T. Late Cutaneous Metastasis Originating from Gastric Cancer with Synchronous Metastasis. Case Rep Gastroenterol. 2019 Jan 1;13(1):95–101.

11. Demircioğlu D, Öztürk Durmaz E, Demirkesen C, Şahin S. Livedoid cutaneous metastasis of signet-ring cell gastric carcinoma. J Cutan Pathol. 2021 Jun 1;48(6):785–8.

12. De Giorgi V, Scarfì F, Trane L, Silvestri F, Maida P, Gori A, et al. Appearance of cutaneous melanoma and subcutaneous metastases in metastatic gastric carcinoma undergoing chemotherapy with oral 5-fluorouracil prodrug. Anticancer Drugs. 2019 Nov 1;30(10):1055–7.

13. Yasuda T, Hayashi S, Nakahata Y, Yasuda Y, Omatsu T, Obora A, et al. Recurrence of cutaneous and lymph node metastases 12 years after radical total gastrectomy for stage IIA gastric cancer. Internal Medicine. 2020;59(11):1387–93.

14. Chen JW, Zheng LZ, Xu DH, Lin W. Extensive cutaneous metastasis of recurrent gastric cancer: A case report. World J Clin Cases. 2021;9(22):6575–81.

15. Isil Kurmus G, Canpolat F, Gönül M, Gökçe A, Pelin Kartal S. Gökçe Isil Kurmus et al.-Cutaneous Metastases of Signet-Ring Cell Gastric Carcinoma: A Case Report Case Report Cutaneous Metastases of Signet-Ring Cell Gastric Carcinoma: A Case Report.

16. Yao S, Zhou P, Li Y, Li Q. Case report: A case of delayed cutaneous metastases from signet-ring cell mixed-type gastric cancer. Front Oncol. 2023;13.

17. Tian L, Ye ZB, Du YL, Li QF, He LY, Zhang HZ. Inflammatory cutaneous metastases originating from gastric cancer: A case report. World J Clin Cases. 2023 Dec 16;11(35):8411–5.

18. Makino H, Naito K, Tsuruta A, Kan K, Toda S, Yoshimura N, et al. [A case of complete remission of metastatic skin carcinoma (erythema type) from advanced gastric cancer by CDDP administration]. Gan To Kagaku Ryoho. 1993 Nov;20(14):2225–8.

19. Tajima H, Matsuki N, Takeda T, Horichi H, Kumaki T, Shima K. [A case of cutaneous and brain metastasis of gastric carcinoma, treated effectively by chemotherapy with CDDP, MMC, etoposide and 5’-DFUR]. Gan To Kagaku Ryoho. 1994 Nov;21(15):2659–62.

20. Matsuoka T, Hosaka S, Inada K, Kawamoto S. [A case of delayed subcutaneous metastases of gastric carcinoma effectively treated with S-1 plus cisplatin chemotherapy]. Gan To Kagaku Ryoho. 2013 Nov;40(12):2262–4.

21. Mezawa S, Homma H, Doi T, Akiyama T, Murakami K, Kogawa K, et al. [A case of gastric carcinoma with multiple skin, bone, and bilateral ovary metastasis; effective treatment by chemotherapy]. Gan To Kagaku Ryoho. 2003 Nov;30(12):1973–5.

22. Morita J, Aoyama T, Amano S, Maezawa Y, Sawazaki S, Numata M, et al. [A Case of Long-Term Survival of Cutaneous Metastasis from Primary Gastric Cancer]. Gan To Kagaku Ryoho. 2017 Nov;44(12):1393–5.

23. Matsuoka K, Hada M, Ohmori H, Yajima A, Nadaya T, Watanabe H, et al. A long-term survival case with recurrent esophageal adenosquamous carcinoma. Int Cancer Conf J. 2021 Jul 14;10(3):191–6.

24. Menghani S V, Barbosa A, Sagerman P, Beal MW, Scott A. Gastric Cardia Adenocarcinoma with Metastasis to the Scalp: A Case Report. Cureus. 2020 Jan 27;

25. Sai K, Aomatsu N, Sakano Y, Okada T, Yonemitsu K, Fukui Y, et al. [A Case of Advanced Gastric Cancer-Recurrence of Skin Metastasis Nine Years after Surgery and Rectal Stenosis Due to Peritoneal Dissemination Ten Years Later]. Gan To Kagaku Ryoho. 2021 Dec;48(13):1865–7.

26. Hao W, Chang R, Liu J, Wang Y, Ren M, Xin K, et al. Case report: A case of advanced gastric cancer with multiple skin metastases, with significant relief from immunotherapy. Front Immunol. 2024 Feb 29;15.

27. Zhang RY, Zhu SJ, Xue P, He SQ. Cutaneous metastasis from esophageal squamous cell carcinoma: A case report. World J Clin Cases. 2022 May 16;10(14):4676–83.

28. Ryu HR, Lee DW, Choi HJ, Kim JH, Ahn H. Scalp metastasis of advanced gastric cancer. Arch Craniofac Surg. 2021 Jun 20;22(3):157–60.

29. Han L, Pan C, Ni Q, Yu T. Case Report: Herceptin as a Potentially Valuable Adjuvant Therapy for a Patient With Human Epidermal Growth Factor Receptor 2-Positive Advanced Esophageal Squamous Cell Carcinoma. Front Oncol. 2021 Feb 1;10.

30. Katayama T, Ishii T, Tono T, Okubo Y, Shinozaki K, Kawasaki Y, et al. [Gastric Cancer Diagnosed with Metastasis of the Navel(Sister Mary Joseph’s Nodule) - A Case Report]. Gan To Kagaku Ryoho. 2016 Nov;43(12):1905–7.
